# LcERF19, an AP2/ERF transcription factor from *Litsea cubeba*, positively regulates geranial and neral biosynthesis

**DOI:** 10.1093/hr/uhac093

**Published:** 2022-04-22

**Authors:** Minyan Wang, Ming Gao, Yunxiao Zhao, Yicun Chen, Liwen Wu, Hengfu Yin, Jiahui Yang, Shifa Xiong, Siqi Wang, Jue Wang, Yang Yang, Jia Wang, Yangdong Wang

**Affiliations:** State Key Laboratory of Tree Genetics and Breeding, Chinese Academy of Forestry, Beijing, 100091, China; Research Institute of Subtropical Forestry, Chinese Academy of Forestry, Hangzhou, Zhejiang, 311400, China; State Key Laboratory of Tree Genetics and Breeding, Chinese Academy of Forestry, Beijing, 100091, China; Research Institute of Subtropical Forestry, Chinese Academy of Forestry, Hangzhou, Zhejiang, 311400, China; State Key Laboratory of Tree Genetics and Breeding, Chinese Academy of Forestry, Beijing, 100091, China; Research Institute of Subtropical Forestry, Chinese Academy of Forestry, Hangzhou, Zhejiang, 311400, China; State Key Laboratory of Tree Genetics and Breeding, Chinese Academy of Forestry, Beijing, 100091, China; Research Institute of Subtropical Forestry, Chinese Academy of Forestry, Hangzhou, Zhejiang, 311400, China; State Key Laboratory of Tree Genetics and Breeding, Chinese Academy of Forestry, Beijing, 100091, China; Research Institute of Subtropical Forestry, Chinese Academy of Forestry, Hangzhou, Zhejiang, 311400, China; State Key Laboratory of Tree Genetics and Breeding, Chinese Academy of Forestry, Beijing, 100091, China; Research Institute of Subtropical Forestry, Chinese Academy of Forestry, Hangzhou, Zhejiang, 311400, China; State Key Laboratory of Tree Genetics and Breeding, Chinese Academy of Forestry, Beijing, 100091, China; Research Institute of Subtropical Forestry, Chinese Academy of Forestry, Hangzhou, Zhejiang, 311400, China; State Key Laboratory of Tree Genetics and Breeding, Chinese Academy of Forestry, Beijing, 100091, China; Research Institute of Subtropical Forestry, Chinese Academy of Forestry, Hangzhou, Zhejiang, 311400, China; State Key Laboratory of Tree Genetics and Breeding, Chinese Academy of Forestry, Beijing, 100091, China; Research Institute of Subtropical Forestry, Chinese Academy of Forestry, Hangzhou, Zhejiang, 311400, China; State Key Laboratory of Tree Genetics and Breeding, Chinese Academy of Forestry, Beijing, 100091, China; Research Institute of Subtropical Forestry, Chinese Academy of Forestry, Hangzhou, Zhejiang, 311400, China; State Key Laboratory of Tree Genetics and Breeding, Chinese Academy of Forestry, Beijing, 100091, China; Research Institute of Subtropical Forestry, Chinese Academy of Forestry, Hangzhou, Zhejiang, 311400, China; State Key Laboratory of Tree Genetics and Breeding, Chinese Academy of Forestry, Beijing, 100091, China; Research Institute of Subtropical Forestry, Chinese Academy of Forestry, Hangzhou, Zhejiang, 311400, China; State Key Laboratory of Tree Genetics and Breeding, Chinese Academy of Forestry, Beijing, 100091, China; Research Institute of Subtropical Forestry, Chinese Academy of Forestry, Hangzhou, Zhejiang, 311400, China

## Abstract

The APETALA2/ETHYLENE RESPONSE FACTOR (AP2/ERF) transcription factors (TFs) are involved in the regulation of specialized terpenoid biosynthesis. However, the AP2/ERF TFs in *Litsea cubeba* have not been characterized and their role in the biosynthesis of terpenoids is unknown. Here, 174 LcAP2/ERF TFs were identified in *L. cubeba* and categorized into four subfamilies: 27 AP2, 7 RAV, 1 Soloist, and 139 ERF. Transcriptomic and qRT-PCR assays both showed that the expression levels of *LcERF19* were similar to that of terpene synthase *LcTPS42* in the pericarp, which is related to the synthesis of geranial and neral in *L. cubeba*. *LcERF19* was further shown to encode a nuclear-localized protein and its expression was strongly induced by jasmonate. Yeast one-hybrid and dual-luciferase assays showed that LcERF19 associated with GCC box elements of the *LcTPS42* promoter and promoted its activity. Transient overexpression of *LcERF19* in *L. cubeba* and overexpression of *LcERF19* in tomato resulted in a significant increase in geranial and neral. Our findings show that LcERF19 enhances geranial and neral biosynthesis through activation of *LcTPS42* expression, which provides a strategy to improve the flavor of tomato and other fruits.

## Introduction

*L. cubeba* (Lour.) Persoon, an aromatic species in the Lauraceae, produces essential oils in the pericarp and is an important raw material for fragrance and medicine biosynthesis. Monoterpenes comprise 94.4-98.4% of *L. cubeba* essential oils (LCEO) and consist of about 41 different types, which include, geranial and neral (78.7–87.4%), limonene, linalool, pinene, eucalyptus, and others [1]. Common monoterpenoids appear as one or several main compounds in aromatic plants and help plants cope with environmental stress and attract pollinators and seed disseminators. [2]. These aromatic and medicinal terpenoids also benefit humans. Citral, a mixture of geranial and neral, is widely used in fragrance, cosmetics, and pharmaceuticals because of its unique and sensitive aroma, antibiotic properties, and oxidative characteristics [3–5]. To improve the production and quality of essential oils with significant commercial value, a better understanding of monoterpenoid metabolism and control mechanisms is necessary.

The monoterpenoid metabolic process has been studied extensively, and is influenced by pathway flux and enzyme biosynthetic step limitation [6, 7]. Transcriptional regulation of enzymes involved in monoterpenoid biosynthesis is one way in which the cell controls pathway flux [8]. Transcription factors (TFs) often coordinate the transcription of multiple metabolic pathways and affect the transcription of genes in the same metabolic pathway [9–11].

The APETALA2/ETHYLENE RESPONSE FACTOR (AP2/ERF) genes play key roles in regulating terpenoid biosynthesis and significantly improve the yield of target terpenoids in plants such as *Catharanthus roseus*, *Artemisia annua*, *Zea mays* (maize), and *Citrus sinensis* (sweet orange) [12–16]. AP2/ERFs are classified into 12 phylogenetic groups [17]. In particular, group IX is implicated in the control of secondary metabolism and is stimulated by defense-related hormones such as jasmonate (JA) [17]. For example, CiERF71 activates the CiTPS16 promotor, which impacts E-geraniol biosynthesis in sweet orange [18]. AaERF2 and AaORA induced ADS or CYP71AV1 promoter activities to positively regulate artemisinin biosynthesis [13, 19]. EREB58 induces sesquiterpene biosynthesis and improves maize disease resistance [15]. These ERFs specifically recognize the cis-regulatory GCC box element to directly or indirectly regulate genes of the secondary metabolic biosynthesis pathway and respond to JA, indicating that the functions of AP2/ERF from different clusters are conserved and interchangeable [20, 21]. All of these JA-regulated ERFs reside in subgroup IXa but not subgroup IXb, and the regulation of plant secondary metabolism by subgroup IXb still remains to be investigated.

As described above, AP2/ERF TFs play significant roles in terpenoid biosynthesis, however, the AP2/ERF genes have not been studied in *L. cubeba*. The candidate AP2/ERF TFs are implicated in the biosynthesis of geranial and neral in *L. cubeba* and were investigated here. Our previous work reported that the expression of *LcTPS42* is favorably connected with the yield of LCEO (geranial and neral) and that LcTPS42 could catalyze geranyl pyrophosphate to produce geraniol. Geraniol was the precursor for the direct biosynthesis of geranial. In the present study, the regulators responsible for the activation of *LcTPS42* expression were further studied. A total of 174 LcAP2/ERFs were identified based on the *L. cubeba* genome database and transcriptome data. LcERF19, a subgroup of IXb AP2/ERF TF, encodes a nuclear-localized protein and directly binds the promoter of *LcTPS42*. Transient expression of LcERF19 in *L. cubeba* and transgenic tomato revealed that *LcERF19* could promote the biosynthesis of geranial and neral. Yeast one-hybrid and dual-luciferase experiments confirmed that LcERF19 activates the *LcTPS42* promoter and potentially regulates *LcTPS42* gene expression. Our study provides a basis for genetic improvement of geranial and neral biosynthesis in *L. cubeba* and genetic engineering of these monoterpenoid products in aromatic plants.

## Results

### Identification of the LcAP2/ERF TFs in *L. cubeba*

**Figure 1:**
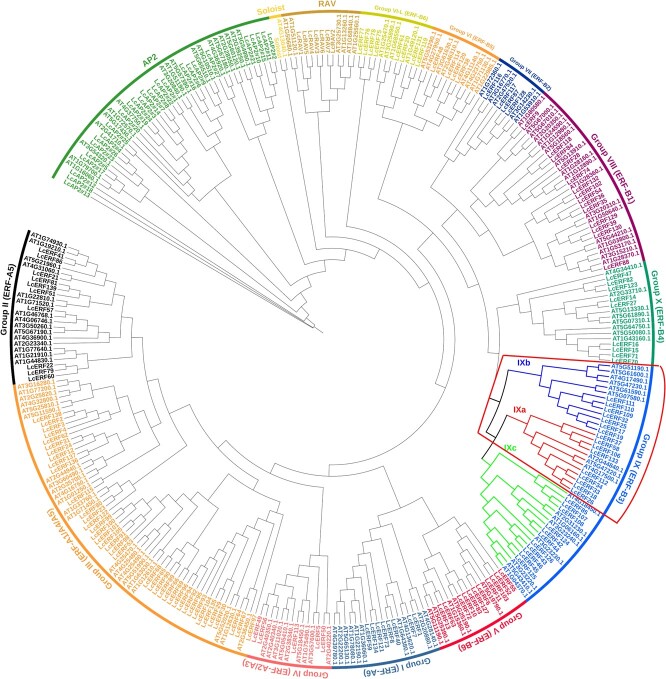
Classification and phylogenetic analysis of the LcAP2/ERF family in *Litsea cubeba*. Different colors represent different groups, and all LcAP2/ERF genes in *L. cubeba* were clustered into subclades based on the priority classification rule of *Arabidopsis thaliana* AP2/ERF genes [17].

To confirm the potential regulation of geranial and neral biosynthesis by AP2/ERF TFs in *L. cubeba*, we first identified the AP2/ERF TFs in *L. cubeba*. According to HMMER and BLAST, 174 genes encoding proteins with the AP2/ERF domain were identified and named based on chromosome location and their phylogenetic classification (Table S2). Phylogenetic analysis indicated that the *L. cubeba* AP2/ERFs could be divided into 27 AP2, 7 RAV, 1 Soloist, and 139 ERF (I to Xb-L) based on the priority classification rule of *Arabidopsis thaliana* AP2/ERF TFs (Fig. 1). Importantly, 16 AP2/ERF members were clustered into the ERF group IX (a + b), which affects the biosynthesis of plant secondary metabolism and is induced by JA [17, 20, 23].

### *LcERF19* and *LcTPS42* have similar expression patterns and were induced by JA in the pericarp

**Figure 2:**
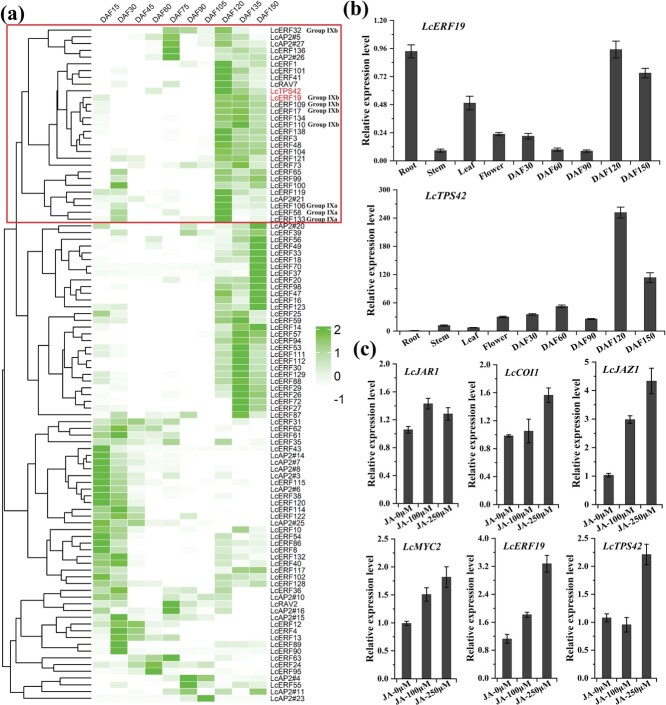
*LcERF19* and *LcTPS42* have similar expression patterns and are induced by JA in the pericarp. (a) Heat map showing expression of the *AP2/ERF* genes and *LcTPS42* in the pericarp based on FPKM values. Only genes with an average FPKM > 2 in at least one of three biological replicates were used to generate the heat map. (b) The expression levels of *LcERF19* and *LcTPS42* in different tissues and pericarp of developmental stages in *Litsea cubeba*. DAF, days after full bloom. (c) MeJA treatment increases the expression levels of JA responsive genes *LcJAR1*, *LcCOI1*, *LcJAZ1*, *LcMYC2*, *LcERF19*, and *LcTPS42* in *L. cubeba* pericarp at 75 DAF. Data are sown as the mean ± standard deviation of three replicates. The *L. cubeba* Ubiquitin-conjugating (UBC) enzyme gene served as the internal control (Lin et al., 2013).

Considering monoterpenoids are mainly produced in the pericarp of *L. cubeba*, the LcAP2/ERF TFs highly expressed in the pericarp are most likely to be involved in monoterpenoid biosynthesis. Previous studies revealed that *LcTPS42* is highly expressed in the pericarp and catalyzes the biosynthesis of geranial and neral (main components of monoterpenoid) in *L. cubeba* [22]. Hence, we included *LcTPS42* in the expression mode analysis. Results from transcriptional sequencing (PRJNA763042) of *L. cubeba* pericarp at different stages of development were used to identify specific AP2/ERF TFs responsible for regulating *LcTPS42* gene expression. Three AP2/ERFs in the IXa subgroup and 5 in the IXb subgroup were coexpressed with *LcTPS42* in the pericarp of *L. cubeba* (Fig. 2a). In particular, *LcERF19* was highly positively correlated with *LcTPS42* gene expression (Fig. 2a), which suggests that LcERF19 may be involved in the regulation of *LcTPS42*.

To further understand the relationship between *LcERF19* and *LcTPS42* gene expression, the expression trends of *LcERF19* and *LcTPS42* were investigated using qRT-PCR. As shown in Fig. 2b, *LcERF19* exhibited the highest expression in roots and the late development of the pericarp. Both *LcERF19* and *LcTPS42* were highly expressed in the later period of pericarp development. To determine whether the expression of *LcERF19* and *LcTPS42* were induced by JA, we selected fruits whose LCEO and JA contents increased rapidly in the pericarp at 75 days after flowering (DAF) [24]. We found that the transcript levels of JA responsive genes *LcJAR1*, *LcCOI1*, *LcJAZ1*, *LcMYC2*), *LcERF19*, and *LcTPS42* all increased with MeJA treatment (Fig. 2c).

### LcERF19 encodes a nuclear-localized protein

**Figure 3:**
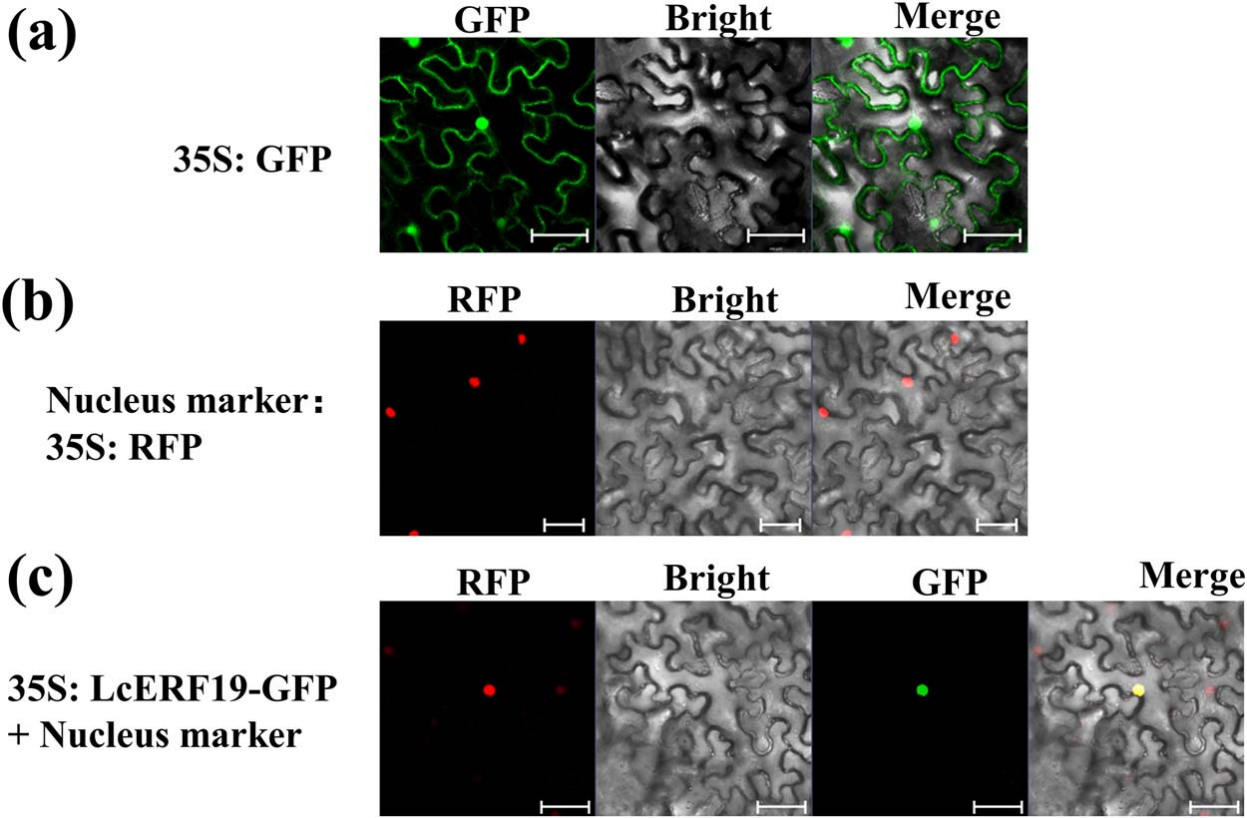
Subcellular localization of LcERF19 in tobacco leaf cells. *Agrobacterium tumefaciens* strains harboring an empty vector (35S:*GFP*), (b) a nuclear marker (35S:*h26-RFP*), or (c) 35S:*LcERF19-GFP* were infiltrated into four-week-old tobacco leaves. Bar = 50 μm.

Transcription factors usually perform transcriptional regulatory functions in the nucleus. The *LcERF19* coding sequence was fused in-frame with *GFP* and transiently expressed in tobacco leaves using *Agrobacterium*  *tumefaciens*-mediated transformation. We observed that free GFP was localized to both the cytoplasm and nucleus of tobacco cells (Fig. 3a) whereas the nuclear marker (Fig. 3b) and LcERF19-GFP colocalized in the nucleus. The results showed that LcERF19 is a nuclear-localized protein (Fig. 3c).

### LcERF19 bound and activated the *LcTPS42* promoter

**Figure 4:**
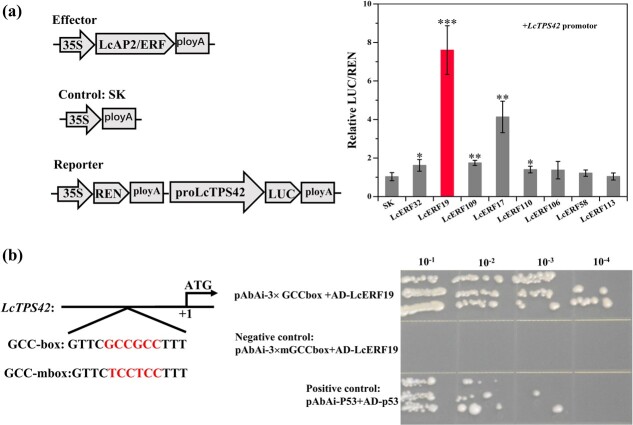
LcERF19 binds to the promoter of *LcTPS42* and activates its expression. (a) Screening of candidate TFs by the dual-LUC assay. The expression levels of AP2/ERFs were significantly increased compared with the control (highlighted in red). Data are shown as the mean ± standard deviation of three replicates (^***^P < 0.001, ^**^P < 0.01; ^*^P < 0.05). (b) LcERF19 binds to the GCC-box regions in the *LcTPS42* promoter. Three repeats of the GCC-box element were used as baits (pAbAi-3 × mGCC-box). Yeast cells coexpressing pGADT7-LcERF19 and the pAbAi-3 × mGCC-box from the *LcTPS42* promoter were cultured for 5 days at 30°C in selective medium (SD/−Trp/-Ura containing 600 ng/mL AbAi).

Our results demonstrated that 3 AP2/ERFs in the IXa subgroup and 5 in the IXb subgroup were coexpressed with *LcTPS42* in the pericarp of *L. cubeba* (Fig. 2a), indicating that these TFs may regulate the expression of *LcTPS42*. We used a dual-LUC assay to determine if these TFs could regulate *luciferase* gene expression driven by the *LcTPS42* promoter. All 8 AP2/ERF transcription factors were expressed from the pGreenII62-SK vector and the *LcTPS42* promoter was placed upstream of *luciferase* in the pGREENII0800-LUC vector. Our results demonstrated that LcERF32, LcERF19, LcERF109, LcERF17, and LcERF110 could significantly activate the *LcTPS42* promotor compared with the control (Fig. 4a). LcERF19, a member of the IXb subgroup, was chosen for additional assays due to its particularly high activation of the *LcTPS42* promoter.

AP2/ERF subgroup IX TFs play a key role in regulating plant secondary metabolism through interacting with a GCC-box region in promoters [14, 15, 18, 21]. We noticed a GCC-box element in the *LcTPS42* promoter and subsequently performed a Y1H assay to determine if LcERF19 could physically associate with the *LcTPS42* promoter. The Y1H assay revealed that LcERF19 interacted with the three tandem duplicates of the GCC-box element in the *LcTPS42* promoter. Yeast expressing *LcERF19* and the *AUR1* gene driven by the wild-type *LcTPS42* promoter were able to grow on media containing AbA whereas mutation of the GCC-box in the *LcTPS42* promoter prevented yeast growth.

### LcERF19 promotes geranial and neral biosynthesis in *L. cubeba*

**Figure 5:**
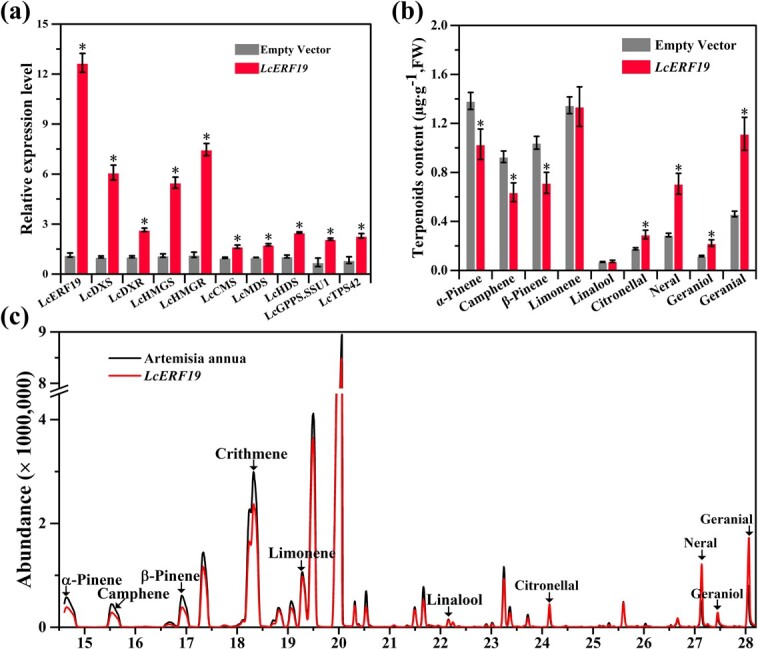
Transient overexpression of *LcERF19* promotes monoterpene biosynthesis and the expression of related genes in *Litsea cubeba*. (a) Expression levels of monoterpenoid biosynthesis pathway genes. (b-c) Contents of monoterpenoids in sterile seedlings overexpressing *LcERF19* determined by GC–MS and sterile *L. cubeba* leaves infected with *Agrobacterium tumefaciens* that contained *LcERF19* driven by the 35S promoter. Data are shown as the mean ± standard deviation of three replicates (^*^P < 0.05).

We investigated whether LcERF19 could increase monoterpenoid yield in *L. cubeba* by activating the *LcTPS42* promoter. Because stable transformation of *L. cubeba* is challenging, an efficient and simple transient expression assay was used to investigate the function of LcERF19. After transient expression of *LcERF19*, we detected a 12.5-fold increase in *LcERF19* expression relative to the control (Fig. 5a). Transient expression of *LcERF19* also revealed that the expression levels of monoterpenoid biosynthesis-related genes *LcDXS*, *LcDXR*, *LcHMGS*, *LcHMGR*, *LcCMS*, *LcMDS*, *LcHDS*, *LcGPPS.SSU1*, and *LcTPS42* were significantly increased compared with the control (Fig. 5a). In addition, the increase in monoterpenoid biosynthesis-related gene expression was positively correlated with monoterpenoid content (Fig. 5b-c), and the contents of primary LCEO components, geranial and neral, increased by 242.02% and 266.72%, respectively (Fig. 5b-c). These findings suggest that LcERF19 enhances geranial and neral biosynthesis.

### LcERF19 promotes geranial and neral biosynthesis in tomato [conserved regulation of terpenoids biosynthesis in plants]

**Figure 6:**
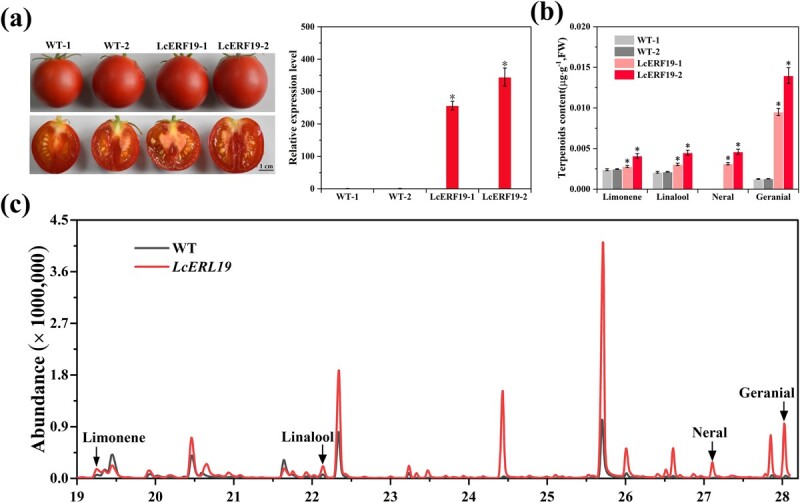
Overexpression of *LcERF19* induces geranial and neral accumulation in tomato fruits. (a) Phenotypes of transgenic tomato fruits and the expression levels of *LcERF19*. WT-1 and WT-2 represent the two wild-type lines and LcERF19–1 and LcERF19–2 represent two independent *LcERF19* overexpression lines. (b-c) Monoterpenoid contents in transgenic tomato fruits 10 d after the breaker stage. Data are shown as the mean ± standard deviation of three replicates (^*^P < 0.05).

To further confirm the conserved role of *LcERF19* in monoterpenoid biosynthesis, we overexpressed *LcERF19* in tomato—an ideal crop to produce terpenoids. A total of 13 transgenic tomato lines were obtained, and we selected tomato fruit from two independent transgenic lines with the highest expression levels of *LcERF19* (LcERF19–1 and LcERF19–2) and two wild-type lines (WT-1 and WT-2) for volatile analysis and qRT-PCR (Fig. S1; Fig. 6a). The results demonstrated that overexpression of *LcERF19* resulted in significantly increased limonene, linalool, neral, and geranial content in transgenic lines compared to WT tomato fruit (Fig. 6b-c). Compared to the control, the content of geranial in the fruit of the LcERF19–2 transgenic tomatoes increased from 0.0012 μg·g^−1^ to 0.0140 μg·g^−1^ (11.7 fold), and the content of neral increased from 0 μg·g^−1^ to 0.0046 μg·g^−1^ (Fig. 6b-c). These findings suggest that *LcERF19* promotes geranial and neral biosynthesis in tomato plants.

## Discussion

### LcERF19 belongs to APT/ERF subgroup IXb and directly binds and activates the *LcTPS42* promotor

The AP2/ERF family is involved in defense signaling regulated by the plant hormones jasmonate, ethylene, and salicylic acid while also regulating the synthesis of specialized compounds [23, 25–28]. LCEO is mainly composed of specialized aromatic monoterpenoid metabolites, including geranial, neral, limonene, linalool, etc. The yield and content of these terpenoids determine the economic value of LCEO [1]. We identified 174 AP2/ERF TFs in the *L. cubeba* genome, which included members of the IX subgroup:9 IXa, 7 IXb, and 11 IXc member genes. The expression of 3 IXa and 5 IXbsubgroup members was positively correlated with that of *LcTPS42*, and these genes were highly expressed in the later phases of fruit peel growth (Fig. 2a). These findings indicated that these TFs may be important to *L. cubeba* terpenoid biosynthesis control.

The IXa and IXb subgroups contain the same basic AP2/ERF domain, recognize specific cis-regulatory elements (GCC-box element), and also share a CMIX2 motif. The CMIX2 motif is a hypothetical acidic region and may play a role as a transcriptional activation domain [17]. In particular, the IXa subgroup has emerged as a broad-spectrum JA-responsive transcriptional regulator in plant specialized metabolite pathways [20, 21]. Examples include ORCA2-ORCA6 regulation of monoterpenoid indole alkaloids in *Catharanthus roseus* [20, 23], GAME9 regulation of steroidal glycoalkaloids in tomato and potato [16], ERF189 regulation of nicotine in tobacco [14], and AaERF2 and AaORA regulation of artemisinin in * Artemisia annua* [13, 19]. However, research on the regulation of plant-specific metabolites by subgroup IXb is lacking. In this study, we observed that 3 IXa and 5 IXb genes were coexpressed with *LcTPS42* (Fig. 2a). Interestingly, the activation effect of 5 IXb TFs (LcERF32, LcERF19, LcERF109, LcERF17, LcERF110) on the *LcTPS42* promoter was stronger than that of IXa subgroup members. The activation effect of LcERF19 was particularly strong, resulting in a 7.6-fold increase in *LcTPS42* promoter activity compared to the control treatment (Fig. 4a). These IXb TFs are similar to the *C. roseus* ORCA TFs, which activate terpenoid biosynthesis pathway genes in a redundant manner [20, 23]. Our results showed that LcERF19 directly binds and activates the *LcTPS42* promotor, and it will be crucial to investigate the control of other ERF family members on specialized metabolite biosynthesis in the future. In addition to the role that IXa subgroup members play in secondary metabolite production, although we have not identified IXa subgroup members that can regulate *LcTPS42* gene expression, IXa subgroup members may also be implicated in the control of other *L. cubeba* terpenoid genes. We also found that LcERF19 recognizes and binds the GCC-box element in the *LcTPS42* promoter. The binding elements are typical binding sites of subgroup IX and the ERF TF subfamily, suggesting that other ERF subfamily members may also regulate terpenoid biosynthesis by binding to this site.

### LcERF19 induces the expression of terpenoid metabolic genes and functions as a positive regulator for terpenoid biosynthesis engineering

In plants, members of the IX subgroup induce the expression of terpenoid biosynthesis genes and improve terpenoid yield and quality [20, 23]. Overexpression of AaORA induces the expression of multiple terpenoid biosynthesis genes and increases artemisinin content [19, 29]. AaERF1 and AaERF2 bind and activate *A. annua* ADS and CYP71AV1 promoters to increase their expression and increase artemisinin content [13]. Similarly, in this study, we found that transient overexpression of *LcERF19* increased the expression of genes involved in the geranial and neral biosynthesis pathways. Geranial and neral contents were increased in tomato fruit overexpressing *LcERF19*. Our results demonstrate that *LcERF19*, a member of subgroup IXb, positively regulates geranial and neral biosynthesis.

Although *LcERF19* overexpression increased geranial and neral contents, we noticed an unexpected decrease in pinene and camphene contents. This may be because the metabolic flow of this monoterpene biosynthesis pathway is relatively stable. *LcERF19* greatly improves the expression of *LcTPS42* and requires more substrates to synthesize geranial and neral, resulting in a reduction in the content of other terpenoids. Similarly, *A. annua* AaTCP15 increased the conversion efficiency of dihydroartemisinic acid to artemisinin by activating the ALDH1 promoter, resulting in increased artemisinin content and decreased dihydroartemisinic acid content in AaTCP15 transgenic plants [30]. However, this is favorable for the genetic improvement of citral production (geranial and citral) with fewer other terpenoids. In addition, overexpression of *LcERF19* in tomato fruit contributed to a significant improvement in citral content, suggesting that LcERF19’s role in promoting citral accumulation is conserved. These findings demonstrate that LcERF19 is an essential key regulator that could be exploited to increase citral production and quality in *L. cubeba*.

## Conclusion

We functionally identified that LcERF19, a member of the IXb subgroup of AP2/ERF TFs, controls geranial and neral biosynthesis. Overexpression of *LcERF19* in *L. cubeba* and tomato significantly increased the contents of geranial and neral. Furthermore, the expression profile of LcERF19 in the pericarp was similar to that of *LcTPS42*, and both were induced by JA. *LcERF19* could also directly bind and activate the *LcTPS42* promoter. Our results indicated that LcERF19 promotes geranial and neral biosynthesis, likely through the activation of *LcTPS42* expression. In the future, more research will be needed to identify and characterize AP2/ERF TF interaction partners and elucidate their involvement in regulating terpenoid metabolism. In addition, we are developing a stable genetic transformation system for *L. cubeba* to express these TFs.

## Materials and methods

### Identification of AP2/ERF gene family members in *L. cubeba* and phylogenetic analysis

The AP2/ERF domain was acquired from the PFAM database. TBtools was used to extract LcAP2/ERF genes from the *L. cubeba* genome [31]. The homologs of *Arabidopsis* AtAP2/ERF transcription factors were downloaded from the PlantTFDB 5.0. The AtAP2/ERF TFs were then used as a query in TBtools to scan the *L. cubeba* protein dataset, using an E-value limit of 1e-5 and a 45% cutoff. Putative LcAP2/ERF proteins were obtained by analyzing HMMER and BLAST results and manually deleting duplicated sequences. NCBICDD, SMART, and PFAM databases were used to double-check the predicted LcAP2/ERF genes [32]. Finally, 174 LcAP2/ERF TFs were identified in the *L. cubeba* genome. The LcAP2/ERFs sequences were aligned using MAFFT, and a phylogenetic tree was constructed using PhyML 3.0 with the default parameters [33].

### Dual-luciferase (dual-LUC) assay

Dual-LUC assays were used to detect LcERF19-mediated activation of *LcTPS42* promoters. TFs were subcloned into a pGreenII62-SK plasmid (effectors) and *LcTPS42* promoters were subcloned upstream of the *luciferase* reporter gene in a pGreenII0800-LUC plasmid (reporters). The empty pGreenII62-SK plasmid was used as a negative control for the effector. Effectors and reporters were individually transformed into *Agrobacterium* strain GV3101. Cultures of individual *Agrobacterium* strains were incubated with shaking overnight. The next day, cultures were pelleted and suspended in infection solution (10 mM MES, 10 mM MgCl2, 200 mM acetosyringone, pH 5.7) and adjusted to an OD600 of1.6. Agrobacterium suspensions in infection solution were then incubated for 4 h at 23–26°C. *Agrobacterium* suspensions containing effectors and reporters were mixed in a 1:1 ratio and infiltrated into the leaves of 30-days-old tobacco plants by needleless syringes. Plants were grown at 26°C for 40–72 h with a 16 h light/8 h dark photoperiod. LUC and REN enzymatic activities were analyzed using a luciferase assay kit (Promega, USA).

### Subcellular localization

*Agrobacterium* strain GV3101 was transformed with a pCAMBIA1300S plasmid containing *LcERF19* fused in-frame with *GFP. Agrobacterium* was prepared for infiltration by pelleting and suspending an overnight culture in infection solution (10 mM MES, 10 mM MgCl2, 200 mM acetosyringone, pH 5.7) and adjusting the concentration to an OD600 of 0.8. The suspension was then incubated for 4 h at 23–26°C and infiltrated into the leaves of 30-day-old tobacco plants. Subcellular protein localization was analyzed at 40–72 h after infiltration using a laser scanning microscope (ZEISS LSM 880, Germany). OsRed was used as a positive control for nuclear localization.

### Yeast one-hybrid (Y1H) assay

Y1H assays were performed as described by Wang et al. (2022)^24^. The *LcERF19* gene was subcloned into the pGADT7 plasmid to obtain pGADT7-prey, while three tandem repeats of GCC-box were subcloned into the pAbAi plasmid to obtain pAbAi-bait. The pAbAi-bait cleaved with the reagent BstBI (NEB, USA) and then cotransformed into yeast with the pGADT7-prey. Transformed yeast were plated on SD/-Trp/-Ura agar plates supplemented with 600 ng/mL of AbA.

### RNA extraction and quantitative real-time PCR (qRT-PCR)

For qRT-PCR analysis, different tissues and fruits of different development stages of *L. cubeba* were harvested from farms in Zhengjiang, China (30°27′94′′N, 119°58′ 43′′E). The harvested tissues were immediately snap-frozen in liquid nitrogen and stored at −80°C. RNA extraction and reverse transcription followed methods described by Zhao et al. (2020) [34]. qRT-PCR was conducted using the TB Green® Premix Ex Taq™ II kit and ABI PRISM 7500 instrument. All primers are summarized in Table S1.

### Transient overexpression of *LcERF19* in *L. cubeba*

*Agrobacterium* strain LBA4404 that contained *LcERF19* in pCAMBIA1300S or the *pCambia1300S* empty vector was used for the transient overexpression assays following Wang et al. (2022)^24^. Sterile leaves of *L. cubeba* seedlings infected with *A. tumefaciens* were placed on MS medium and cultured at 25–27°C with a photoperiod of 16 h light/8 h dark for 40–72 h. Leaves were gathered and stored at −80°C for volatile and qRT-PCR analysis. The volatiles were analyzed using GC–MS [34].

### Transgenic overexpression of *LcERF19* in tomato

The recombinant vector that contained *LcERF19* (*pCAMBIA1300S-LcERF19*) was transformed into *Agrobacterium* strain LBA4404 for transient expression in tomato according to the protocol reported by Chetty et al. (2013) [35]. Seven-day-old cotyledon explants were infected with *Agrobacterium* and cultured on KCMS medium for 40–48 h in the dark. The cotyledon explants were then subcultured in callus induction 2Z medium supplemented with 4 mg/L Hygromycin B and 180 mg/L Timentin to induce callus growth. After 30–45 d, the calluses were transferred to 0.2Z media to induce shoot formation, and the shoots were placed in MS medium to induce root formation. Following the formation of roots, the seedlings were planted in soil and cultivated in a greenhouse. Genomic PCR using primers specific for both the Hygromycin gene and the *LcERF19* gene was performed to verify positive transformants. Samples were harvested from two independent transgenic tomato lines with high *LcERF19* expression and stored at −80°C for volatile and qRT-PCR analysis.

## Acknowledgments

This work was supported by the National Natural Science Foundation of China (31370576).

## Author contributions

CYC, WYD, and GM: Conceptualization, Funding acquisition, Supervision**,** Writing-review & editing. WMY, ZYX, YHF, and WLW: Writing-original draft, Investigation, Methodology, Writing-review & editing. YJH, WSQ, XSF, YY, and WJ: Writing- Proofreading of initial manuscript format and spelling, Investigation.

## Data availability

All data are presented inside the manuscript and its supplementary data.

## Competing interests

The authors declare no competing interests.

## Supplementary data

Supplementary data is available at *Horticulture Research * online.

## Supplementary Material

Web_Material_uhac093

## References

[1] Si L, Chen Y, Han X et al. Chemical composition of essential oils of *Litsea cubeba* harvested from its distribution areas in China. Molecules. 2012;17:7057–66. 22683894 10.3390/molecules17067057 PMC6268156

[2] Pichersky E, Raguso RA. Why do plants produce so many terpenoid compounds? New Phytol. 2018;220:692–702. 27604856 10.1111/nph.14178

[3] Christianson DW . Structural and chemical biology of terpenoid cyclases. Chem Rev. 2017;117:11570–648. 28841019 10.1021/acs.chemrev.7b00287 PMC5599884

[4] Lei Y, Fu P, Jun X et al. Pharmacological properties of geraniol - a review. Planta Med. 2019;85:48–55. 30308694 10.1055/a-0750-6907

[5] Sharma S, Habib S, Sahu D et al. Chemical properties and therapeutic potential of citral, a monoterpene isolated from lemongrass. Med Chem. 2021;17:2–12. 31880247 10.2174/1573406416666191227111106

[6] Ashour M, Wink M, Gershenzon J. Biochemistry of terpenoids: monoterpenes, sesquiterpenes, and diterpenes. Biochemistry of Plant Secondary Metabolism. 2010;40:258–303.

[7] Bian G, Deng Z, Liu T. Strategies for terpenoid overproduction and new terpenoid discovery. Curr Opin Biotechnol. 2017;48:234–41. 28779606 10.1016/j.copbio.2017.07.002

[8] Vattekkatte A, Garms S, Brandt W et al. Enhanced structural diversity in terpenoid biosynthesis: enzymes, substrates, and cofactors. Org Biomol Chem. 2018;16:348–62. 29296983 10.1039/c7ob02040f

[9] Rushton PJ, Somssich IE, Ringler P et al. WRKY transcription factors. Trends Plant Sci. 2010;15:247–58. 20304701 10.1016/j.tplants.2010.02.006

[10] Dubos C, Stracke R, Grotewold E et al. MYB transcription factors in *Arabidopsis*. Trends Plant Sci. 2010;15:573–81. 20674465 10.1016/j.tplants.2010.06.005

[11] Zhang F, Fu X, Lv Z et al. A basic leucine zipper transcription factor, AabZIP1, connects abscisic acid signaling with artemisinin biosynthesis in *Artemisia annua*. Mol Plant. 2015;8:163–75. 25578280 10.1016/j.molp.2014.12.004

[12] Menke FL, Champion A, Kijne JW et al. A novel jasmonate- and elicitor-responsive element in the periwinkle secondary metabolite biosynthetic gene Str interacts with a jasmonate- and elicitor-inducible AP2-domain transcription factor, ORCA2. EMBO J. 1999;18:4455–63. 10449411 10.1093/emboj/18.16.4455 PMC1171520

[13] Yu ZX, Li JX, Yang CQ et al. The jasmonate-responsive AP2/ERF transcription factors AaERF1 and AaERF2 positively regulate artemisinin biosynthesis in *Artemisia annua* L. Mol Plant. 2012;5:353–65. 22104293 10.1093/mp/ssr087

[14] Shoji T, Hashimoto T. DNA-binding and transcriptional activation properties of tobacco NIC2-locus ERF189 and related transcription factors. Plant Biotechnol J. 2012;29:35–42.

[15] Li S, Wang H, Li F et al. The maize transcription factor EREB58 mediates the jasmonate-induced production of sesquiterpene volatiles. Plant J. 2015;84:296–308. 26303437 10.1111/tpj.12994

[16] Cárdenas PD, Sonawane PD, Pollier J et al. GAME9 regulates the biosynthesis of steroidal alkaloids and upstream isoprenoids in the plant mevalonate pathway. Nat Commun. 2016;7:10654. 26876023 10.1038/ncomms10654 PMC4756317

[17] Nakano T, Suzuki K, Fujimura T et al. Genome-wide analysis of the ERF gene family in *Arabidopsis* and rice. Plant Physiol. 2006;140:411–32. 16407444 10.1104/pp.105.073783 PMC1361313

[18] Li X, Xu Y, Shen S. Transcription factor CitERF71 activates the terpene synthase gene *CitTPS16* involved in the synthesis of E-geraniol in sweet orange fruit. J Exp Bot. 2017;68:4929–38. 28992329 10.1093/jxb/erx316 PMC5853461

[19] Lu X, Zhang L, Zhang F et al. AaORA, a trichome-specific AP2/ERF transcription factor of *Artemisia annua*, is a positive regulator in the artemisinin biosynthetic pathway and in disease resistance to *Botrytis cinerea*. New Phytol. 2013;198:1191–202. 23448426 10.1111/nph.12207

[20] Paul P, Singh SK, Patra B et al. Mutually regulated AP2/ERF gene clusters modulate biosynthesis of specialized metabolites in plants. Plant Physiol. 2020;182:840–56. 31727678 10.1104/pp.19.00772 PMC6997685

[21] Shoji T, Yuan L. ERF gene clusters: working together to regulate metabolism. Trends Plant Sci. 2021;26:23–32. 32883605 10.1016/j.tplants.2020.07.015

[22] Chen YC, Li Z, Zhao YX et al. The *Litsea* genome and the evolution of the laurel family. Nat Commun. 2020;11:1675. 32245969 10.1038/s41467-020-15493-5 PMC7125107

[23] Paul P, Singh SK, Patra B et al. A differentially regulated AP2/ERF transcription factor gene cluster acts downstream of a MAP kinase cascade to modulate terpenoid indole alkaloid biosynthesis in *Catharanthus roseus*. New Phytol. 2017;213:1107–23. 27801944 10.1111/nph.14252

[24] Wang MY, Jiao YL, Gao M et al. Phytohormone and transcriptome of pericarp reveals jasmonate and LcMYC2 are involved in neral and geranial biosynthesis in Litsea cubeba. Ind Crop Prod. 2022;177:114423.

[25] Huang Q, Sun M, Yuan T et al. The AP2/ERF transcription factor SmERF1L1 regulates the biosynthesis of tanshinones and phenolic acids in *salvia miltiorrhiza*. Food Chem. 2019;274:368–75. 30372953 10.1016/j.foodchem.2018.08.119

[26] Xie Z, Nolan TM, Jiang H et al. AP2/ERF transcription factor regulatory networks in hormone and abiotic stress responses in *Arabidopsis*. Front Plant Sci. 2019;10:228. 30873200 10.3389/fpls.2019.00228 PMC6403161

[27] Yuan L . Clustered ERF transcription factors: not all created equal. Plant Cell Physiol. 2020;61:1025–7. 32392307 10.1093/pcp/pcaa067 PMC7295393

[28] Jiang CX, Fei X, Pan XJ et al. Tissue-specific transcriptome and metabolome analyses reveal a gene module regulating the terpenoid biosynthesis in *Curcuma wenyujin*. Ind Crop Prod. 2021;170:113758.

[29] Ma YN, Xu DB, Li L et al. Jasmonate promotes artemisinin biosynthesis by activating the TCP14-ORA complex in *Artemisia annua*. Sci Adv. 2018;4:eaas9357. 30627665 10.1126/sciadv.aas9357 PMC6317983

[30] Ma YN, Xu DB, Yan X et al. Jasmonate-and abscisic acid-activated AaGSW1-AaTCP15/AaORA transcriptional cascade promotes artemisinin biosynthesis in *Artemisia annua*. Plant Biotechnol J. 2021;19:1412–28. 33539631 10.1111/pbi.13561 PMC8313134

[31] Chen C, Chen H, Zhang Y et al. TBtools: an integrative toolkit developed for interactive analyses of big biological data. Mol Plant. 2020;13:1194–202. 32585190 10.1016/j.molp.2020.06.009

[32] Najafi S, Sorkheh K, Nasernakhaei F. Characterization of the APETALA2/ethylene-responsive factor (AP2/ERF) transcription factor family in sunflower. Sci Rep. 2018;8:11576. 30068961 10.1038/s41598-018-29526-z PMC6070487

[33] Guindon S, Dufayard JF, Lefort V et al. New algorithms and methods to estimate maximum-likelihood phylogenies: assessing the performance of PhyML 3.0. Syst Biol. 2010;59:307–21. 20525638 10.1093/sysbio/syq010

[34] Zhao YX, Chen YC, Gao M et al. Overexpression of geranyl diphosphate synthase small subunit 1 (LcGPPS. SSU1) enhances the monoterpene content and biomass. Ind Crop Prod. 2020;143:111926.

[35] Chetty VJ, Ceballos N, Garcia D et al. Evaluation of four *agrobacterium* tumefaciens strains for the genetic transformation of tomato (*Solanum Lycopersicum* L.) cultivar micro-tom. Plant Cell Rep. 2013;32:239–47. 23099543 10.1007/s00299-012-1358-1

