# Supplementary figures and images for: LcERF19, an AP2/ERF transcription factor from Litsea cubeba, positively regulates geranial and neral biosynthesis
Source: Hortic Res. 2022 Apr 22;9:uhac093. doi: 10.1093/hr/uhac093 (PMC9327096; doi:10.1093/hr/uhac093)

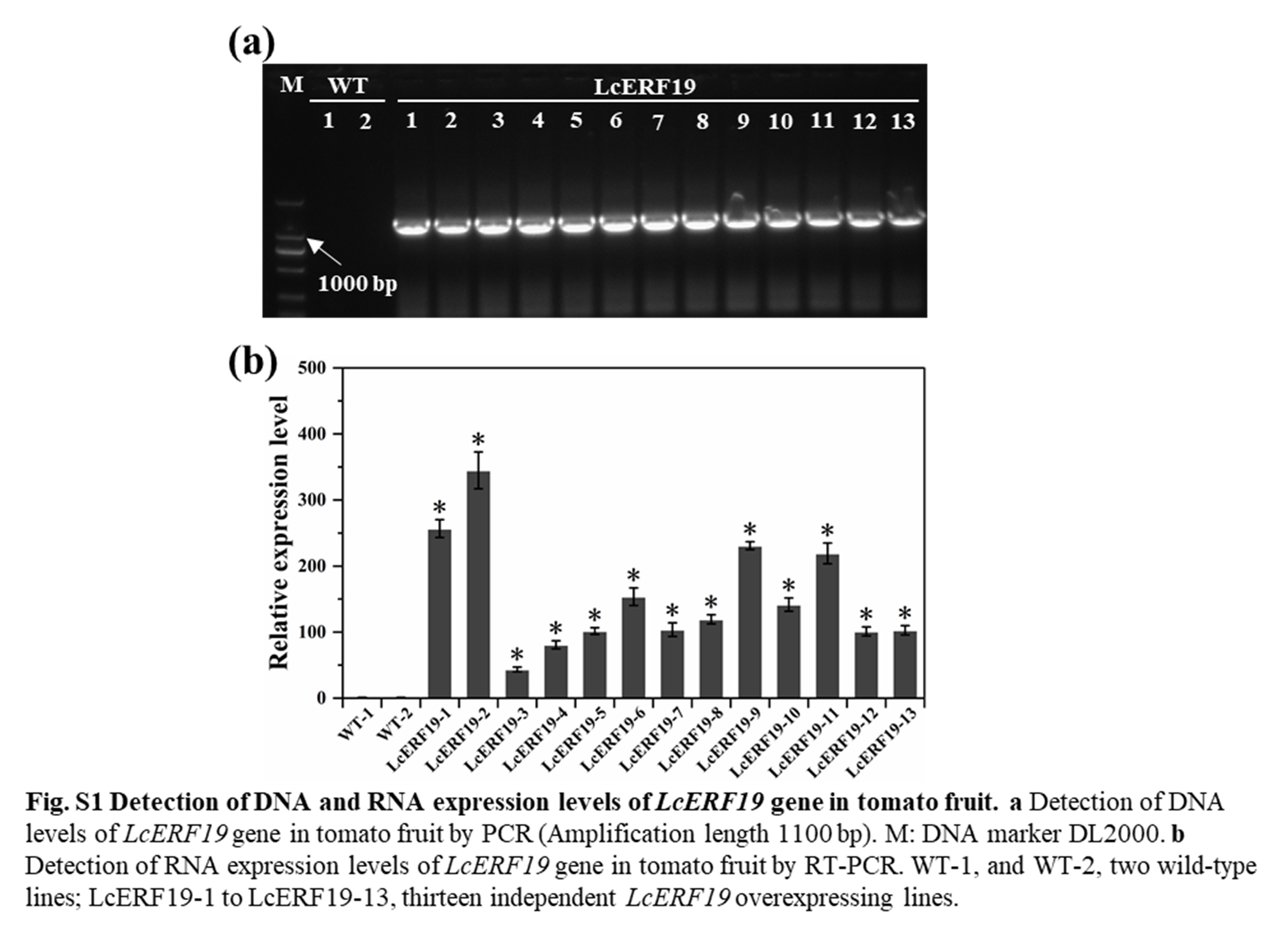

Supplement: Web_Material_uhac093 [file web_material_uhac093.zip › Fig. S1.jpg]
